# Assessment of oxidative stress and trace element dynamics in acute myocardial infarction and heart failure: a focus on zinc, copper, and thiol dynamics

**DOI:** 10.1016/j.clinsp.2025.100755

**Published:** 2025-08-21

**Authors:** Yasin Ali Cimen, Bahadir Taslidere, Ufuk Sarikaya, Metin Demirel, Nusret Acikgoz, Sahabettin Selek

**Affiliations:** a Department of Physiology, Faculty of Medicine, Bezmialem Vakif University, Istanbul, Turkey; b Department of Emergency Medicine, Faculty of Medicine, Bezmialem Vakif University, Istanbul, Turkey; c Department of Medical Biochemistry, Faculty of Medicine, Bezmialem Vakif University, Istanbul, Turkey; d Department of Cardiology, Faculty of Medicine, Bezmialem Vakif University, Istanbul, Turkey

**Keywords:** Acute myocardial infarction (AMI), Copper (Cu), Heart failure (HF), Oxidative Stress, Thiols, Zinc (Zn)

## Abstract

**Background:**

Acute Myocardial Infarction (AMI) and Heart Failure (HF) are the leading global causes of death from Cardiovascular Diseases (CVD). This study aimed to investigate the association of serum Zinc (Zn), Copper (Cu), and thiol levels with CVD and new potential biomarkers.

**Methods:**

The study included serum samples from 150 participants, including 50 AMI patients, 50 HF patients, and 50 healthy individuals. Oxidative stress, trace elements (Cu and Zn), and thiol levels were investigated by automated kits in AMI and HF patients compared to the control group.

**Results:**

T-thiol, N-thiol and Cu levels were higher in the AMI group than in the control group (*p* < 0.05, *p* < 0.01, *p* < 0.01, respectively). Cu and Zn levels were higher in the HF group compared to the control group (*p* < 0.001, *p* < 0.01, respectively). Compared to the HF group, T-thiol (*p* < 0.05), N-thiol (*p* < 0.05), and Zn (*p* < 0.001) levels were higher in the AMI group. By ROC analysis, Zn, Cu and thiol levels showed moderate discriminative power, emphasizing their candidacy as biomarkers.

**Conclusions:**

Despite the limitations of age-related variations and sample size, this study pioneers the comparative analysis of AMI and HF, contributing to the elucidation of the complex connections between Zn, Cu and thiol levels in cardiovascular pathologies. It increases the importance of Zn, Cu and thiol levels in CVD’s status, necessitating further research on this topic with refined methodologies.

## HIGHLIGHTS

• Oxidative and thiol balance is altered in AMI and HF.
• Serum zinc levels are elevated in AMI.
• Zinc and copper have the potential to be biomarkers in AMI and HF.

## Introduction

Cardiovascular Diseases (CVD) were responsible for a third of all deaths in 2021.^1^ Among emergency hospitalizations, Acute Myocardial Infarction (AMI) stands out as the CVD with the highest mortality and morbidity.^2^ AMI often results from atherosclerotic plaque movement, leading to thrombotic events, myocardial necrosis, and potentially progressing to Heart Failure (HF). In conditions like AMI and HF, the heart faces challenges in effective oxygen and nutrient delivery, triggering an increase in reactive oxygen species release.^3^ Despite adaptive responses, oxidative stress levels exceed the body's current capacity. Oxidative stress plays a pivotal role in initiating and advancing atherosclerosis, leading to endothelial dysfunction and impaired Nitric Oxide (NO) and NF-kB-associated signaling. It is suggested that the most important reason for this situation is that accumulated oxidative stress contributes to atherosclerosis and increased plaque susceptibility by reducing endothelial NO synthesis.^4^ This vicious cycle caused by oxidative stress is critical in CVD, where deaths and complications develop.^5^

Various studies have questioned the role of trace elements in the antioxidant defense system. Copper (Cu) and Zinc (Zn), which are essential elements for oxidative balance, have been reported to play a fundamental role in antioxidant and anti-inflammatory aspects.^6^ The pivotal antioxidant enzyme Zinc-Copper Superoxide Dismutase (Cu-Zn SOD) inhibits superoxide toxicity in cells.^7^ Zn deficiency is recognized for its role in constraining immune responses and elevating oxidative stress in chronic diseases.^8^ Cu, another vital trace element, acts as a cofactor for enzymes like superoxide dismutase and cytochrome oxidase. However, an increase in Cu levels can lead to toxicosis, enhancing organic damage through oxidative stress.^9^ In summary, Zn and Cu are crucial in maintaining homeostasis, especially from an antioxidant and anti-inflammatory perspective.^10^ Thiols are organic groups with functional Sulphur and Hydrogen (SH) atoms bonded to the carbon atom in the cysteine Structure (-S-S). Cysteine plays a role in protein structure, signal transduction, molecular stabilization, and antioxidant defence.^11^ Thiols can undergo oxidation reactions with oxidizing agents and form disulfide bonds in the process.^12^ Disruption of thiol-disulfide homeostasis has been reported in the pathogenesis of CVD.^13^

Although AMI and HF, which are different clinical presentations of cardiovascular diseases, share some common pathophysiological mechanisms, they may differ in terms of disease course, cellular responses and biochemical profiles. Therefore, comparative evaluation of how oxidative stress-related parameters such as Zn, Cu and thiol/disulfide homeostasis change in these two patient groups may contribute both to the identification of disease-specific biomarkers and to a better understanding of differentiated pathophysiological processes. Accordingly, in the present study, the authors aimed to examine the effects of Zn, Cu and thiol/disulfide balance on oxidative stress by comparing AMI and HF patients and to reveal the potential of these parameters as discriminative biomarkers.

## Methods

Study adhered to the Helsinki Declaration, approved by Bezmialem Vakif University Non-Interventional Research Ethics Committee (2021/134, 03.05.2021). The present study consisted of a total of 150 individuals, 50 with AMI, 50 with HF, and 50 healthy individuals as a control group. Volunteers gave written consent. No new vascular access; tests from routine blood samples. Participants met the inclusion criteria; exclusions were based on defined criteria.

### Inclusion criteria

Patients included in the study were identified in accordance with the guidelines of the European Society of Cardiology.^14^ The diagnosis of AMI was established based on the 2023 ESC Guidelines for Acute Coronary Syndromes (ACS). Patients in this group presented to the emergency department with chest pain and/or shortness of breath. The diagnosis was confirmed by dynamic elevation in cardiac troponin levels, ischemic changes on electrocardiography (e.g., ST-segment elevation or depression, T-wave inversion), and evidence of ≥50 % coronary artery stenosis on coronary angiography.^15^

HF was diagnosed according to the 2021 ESC Guidelines for the Diagnosis and Treatment of Acute and Chronic Heart Failure. Patients presented with dyspnoea and chest discomfort, and the diagnosis was based on typical clinical findings (such as pulmonary rales, peripheral oedema, or the presence of a third heart sound), along with elevated Brain Natriuretic Peptide (BNP) levels above age-adjusted thresholds. In selected cases, transthoracic echocardiography was used to further support the diagnosis.^16^

The control group comprised individuals aged 18-years or older who presented to the hospital for non-cardiac complaints and were found, upon medical evaluation, to have no acute or chronic medical conditions, no history of cardiovascular disease, and were not on any regular medication. These individuals were considered clinically healthy.

### Exclusion criteria

Individuals under 18-years of age were excluded from all groups. In the AMI group, patients with angiographic stenosis of less than 50 % were not included. In the control group, individuals with any known systemic disease, a history of cardiovascular disease, regular medication use, or abnormal clinical findings were excluded. Furthermore, individuals who did not volunteer to participate or failed to provide written informed consent were also excluded from the study.

### Sample preparation

Blood samples from the cubital vein, obtained as part of routine clinical procedures, were collected in biochemistry tubes without separating gel, centrifuged at 3500 RPM for 10 min and stored at −80 °C. No additional blood samples were collected from the patients, and no new venous access was established for the purposes of this study. Individuals who presented to the emergency department of Bezmialem Vakif University and met the inclusion criteria were included in the study.

## Materials and methods

The method developed by Erel,^17^ was used in the measurement of serum Total Antioxidant Status (TAS) levels. The antioxidant capacity was determined by reading the product at 444 nm in a spectrophotometer device calibrated with Trolox (6‑hydroxy-2,5,7,8-tetramethylchroman-2-carboxylic acid), a vitamin E analogy. The method created by Erel.,^18^ was used to determine serum Total Oxidant Status (TOS) levels. The oxidants present in the sample oxidize the Fe^+2^-o-dianisidine complex to Fe^+3^. The total oxidant capacity was determined by reading the product at 560 nm and 800 nm in the spectrophotometer device calibrated with H_2_O_2_. The Oxidative stress index value was calculated with the formula TOS/TAS ×100.^19^

Serum Zn and Cu levels were assessed using the Rel Assay (Gaziantep, Turkey) Zn measurement kit. For Zn, 5-Br-PAPS chelator induced a red-violet color in the basic environment, measured at 548 nm. Cu measurement involved 3,5-DiBr-PAESA chelator, creating an indigo color in acidic conditions, measured at 572 nm. A Siemens Atellica® CH 930 analyzer ensured accurate readings.

Serum total and native thiol levels were tested by a new procedure developed by Erel. The total thiol content of the sample was measured at 410 nm using the modified Ellman reagent. The native thiol content is subtracted from the total thiol content, and half of the difference obtained gives the number of disulfide bonds.^20^ All these analyses were performed in the research laboratory of Bezmialem Vakif University using a fully automated photometric method (Siemens ADVIA 1800 clinical chemistry analyzer).

As part of the study, patients were categorized into three groups: control, AMI and HF. To assess oxidative stress, trace element levels and thiols balance, serum concentrations of TAS, TOS, Zn, Cu, T-thiol, N-thiol, and disulfide were measured and compared across the groups.

### Statistical analysis

Sample sizes were determined using G Power. Normality was assessed with the Kolmogorov-Smirnov test. One-way ANOVA analyzed normal distribution data, and Kruskal-Wallis handled abnormal distribution. Post-hoc comparisons utilized Tukey and Dunn tests. IBM SPSS v27.0 (IBM Corporation, Armonk, NY, USA) conducted statistical analyses, presenting data in Table 1 (mean ± SD). To account for demographic differences among groups, particularly age, multivariate linear regression analyses were conducted for key biomarkers using group (categorical) and age (continuous) as independent variables. A *p*-value of < 0.05 was considered statistically significant. Cluster heatmaps, generated by MetaboAnalyst v2.0, employed the Euclidean Distance-Ward connection approach. In this project, no artificial intelligence-assisted technologies were used.

**Table 1:** Demographic data and measured parameter results.

Parameters	C	AMI	HF
Age (years)	35.68 ± 11.34	60.18 ± 13.41^c^	67.46 ± 13.01^c^
Gender	36 M, 14 F	38 M, 12 F	31 M, 19 F
TAS (mmol Trolox Eq/L)	1.51 ± 0.08	1.53 ± 0.77^a^	1.59 ± 0.03^c^^,^^g^
TOS (μmol H_2_O_2_ Eq/L)	18.15 ± 4.48	21.77 ± 3.78	19.79 ± 4.57
OSI (mmol Trolox Eq/L)	1.30 ± 0.229	1.46 ± 0.27	1.41 ± 0.36
T-thiol (µmol/L)	253.23 ± 14.58	260.39 ± 16.15^b^^,^^d^	251.54 ± 19.18
N-thiol (µmol/L)	218.93 ± 16.75	226.78 ± 18.17^a^^,^^d^	214.51 ± 21.79
Disulfide (µmol/L)	18.92 ± 3.02	17.06 ± 2.02^b^	19.28 ± 2.73^h^
Zn (µg/dL)	86.40 ± 15.06	93.46 ± 12.27^f^	79.35 ± 23.62^a^
Cu (µg/dL)	118.24 ± 20.34	130.45 ± 16.23^b^	143.50 ± 26.95^c^
Hypertension	‒	13 (26 %)	12 (24 %)
Diabetes mellitus	‒	10 (20 %)	12 (24 %)

Data are presented as mean ± SD. Statistical significance was denoted as follows:

TAS, Total Antioxidant Status; TOS, Total Oxidant Status; OSI, Oxidative Stress Index; T-thiol, Total Thiol; N-thiol, Native Thiol; Zn, Zinc; Cu, Copper; C, Control; AMI, Acute Myocardial Infarction; HF, Heart Failure; F, Female; M, Male.

a *p* < 0.05.

b *p* < 0.01.

c *p* < 0.001 compared to the control group.

d *p* < 0.05.

f *p* < 0.001 compared to the HF group.

g *p* < 0.05.

h *p* < 0.001 compared to the AMI group.

## Results

### Sociodemographic data

Sociodemographic parameters, as presented in Table 1, highlight notable differences among groups, particularly in age. Both AMI and HF groups exhibited significantly higher ages compared to the control group (*p* < 0.001), with no significant age difference observed between the AMI and HF groups (*p* > 0.05).

### Oxidative stress status

**Fig. 1:**
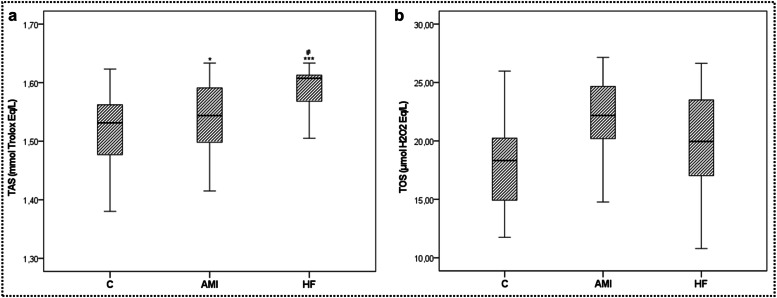
Serum TAS (a) and TOS (b) values of the study groups. Data are presented as mean ± SD (*n* = 50). Statistical significance was denoted as follows: * *p* < 0.05, *** *p* < 0.001 compared to the control group. ## *p* < 0.05 compared to the AMI group. Abbreviations: TAS, Total Antioxidant Status; TOS, Total Oxidant Status; C, Control; AMI, Acute Myocardial Infarction; HF, Heart Failure.

Noteworthy findings emerged in the analysis of TAS. The mean TAS level was significantly higher in the AMI group than the control group (*p* < 0.05) but significantly lower than in the HF group (*p* < 0.01). The HF group exhibited a significantly higher mean TAS level than the control group (*p* < 0.001, Fig. 1a). Serum TOS values, although highest in the AMI group, did not exhibit a significant difference between the groups (*p* > 0.05, Fig. 1b; see Table 1).

### Trace elements: Cu and Zn status

**Fig. 2:**
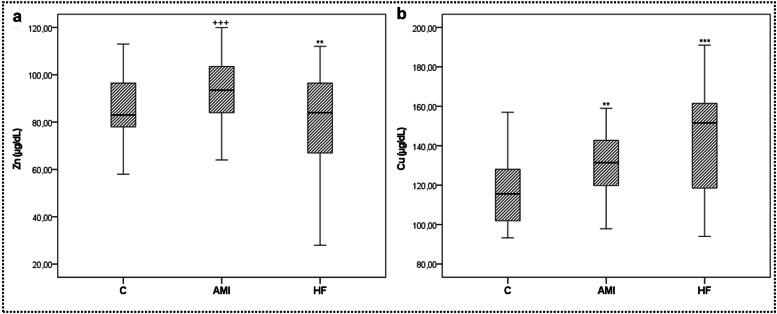
Serum Zn (a) and Cu (b) values of the study groups. Data are presented as mean ± SD (*n* = 50). Statistical significance was denoted as follows: *p < 0.05, ***p < 0.001 compared to the control group. +++*p* < 0.001 compared to the HF group. Abbreviations: Zn, Zinc; Cu, Copper; C, Control; AMI, Acute Myocardial Infarction; HF, Heart Failure.

Mean serum Zn levels were 86.40 µg/dL (±15.06) for the control group, 93.46 µg/dL (±12.27) for the AMI group, and 79.35 µg/dL (±23.62) for the HF group (Table 1). Zn levels were significantly lower in the HF group than in the AMI and control groups (*p* < 0.001, *p* < 0.05 respectively), as illustrated in Fig. 2a.

The mean serum Cu level was 118.24 µg/dL (±20.34) for the control group, 130.45 µg/dL (±16.23) for the AMI group, and 143.50 µg/dL (±26.95) for the HF group (Table 1). Cu concentration was significantly higher in both the AMI (*p* < 0.01) and HF (*p* < 0.001) groups compared to the control group, as depicted in Fig. 2b

### Total and native thiol levels

**Fig. 3:**
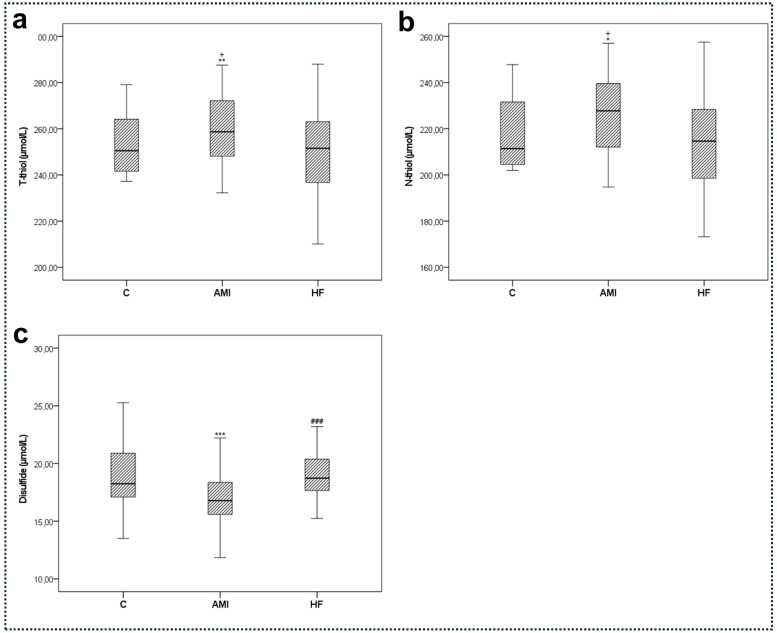
Serum t-thiol (a), N-thiol (b), and disulfide level (c) values of the study groups. Data are presented as mean ± SD (*n* = 50). Statistical significance was denoted as follows: * *p* < 0.05, ** *p* < 0.01, *** *p* < 0.001 compared to the control group. ### *p* < 0.001 compared to the AMI group. + *p* < 0.05 compared to the HF group. Abbreviations: T-thiol, Total Thiol; N-thiol, Native Thiol; C, Control; AMI, Acute Myocardial Infarction; HF, Heart Failure.

Total thiol levels varied among groups, with statistically higher levels in the AMI group compared to the control and HF groups (*p* < 0.01, *p* < 0.05 respectively). Native thiol levels were also significantly higher in the AMI group compared to the control and HF groups (*p* < 0.05, Fig. 3b). Calculated disulfide levels were lower in the AMI group than in the control and HF groups (*p* < 0.01, *p* < 0.001 respectively, Fig. 3c).

Heatmap analysis revealed distinct patterns in oxidative stress and trace element levels across groups. The AMI group exhibited the highest OSI and TOS values, while the control group had the lowest. Cu levels peaked in the HF group and were lowest in controls, while Zn levels showed the opposite trend. N-thiol values were highest in AMI and lowest in HF, TAS peaked in HF and hit its lowest in controls, and T-thiol was highest in AMI and lowest in HF (Supplemental Fig. 1).

**Fig. 4:**
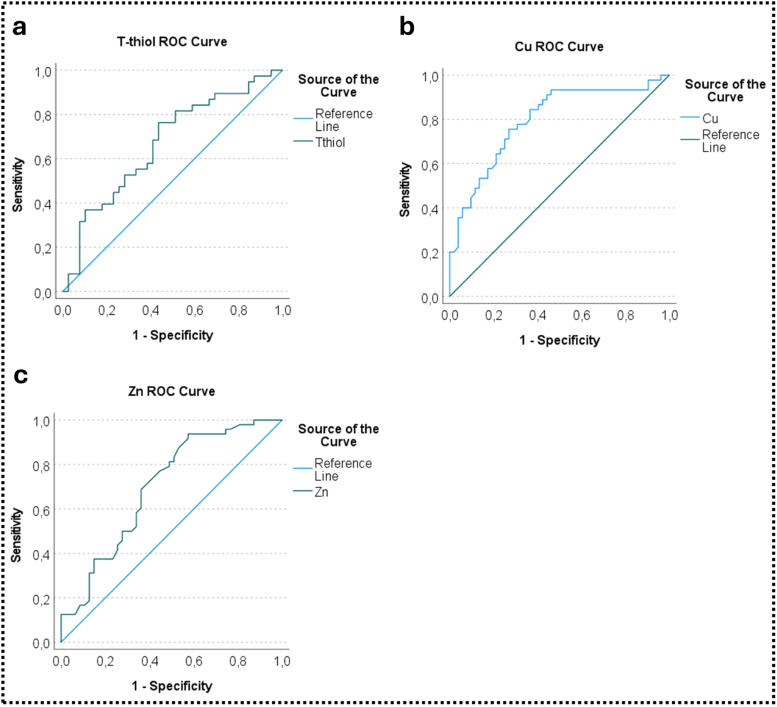
ROC analysis graph of T-thiol (a), Cu (b) and Zn (c) levels in groups. Abbreviations: T-thiol, Total Thiol, Cu, Copper, Zn, Zinc.

Fig. 4a depicts the Receiver Operating Characteristic (ROC) analysis for T-thiol levels in the AMI and HF groups. The Area Under the Curve (AUC) value for Cu was 0.669. The identified cutoff value for T-thiol was 245.48 μmol/L. This threshold exhibited a predictive accuracy for AMI with 68.4 % sensitivity and 59 % specificity (54.7 %‒79.0 %, *p* > 0.05), as summarized in Table 2. In Fig. 4b, the ROC analysis illustrates Cu levels in the HF and control groups, yielding an AUC of 0.796. The determined cutoff value for Cu was 121.1 μg/dL. This threshold demonstrated substantial predictive capabilities for HF, with 75.6 % sensitivity and 73.1 % specificity (70.5 %‒88.6 %, *p* < 0.001), as presented in Table 2. Furthermore, Fig. 4c provides the ROC analysis of Zn levels in the AMI and HF groups. The AUC for Zn in the AMI group was 0.700, with a cutoff value of 84.50 μg/dL. This value exhibited a predictive accuracy for AMI, with 68.8 % sensitivity and 63.8 % specificity (59.4 %‒80.6 %, *p* < 0.01), as outlined in Table 2. These ROC analyses highlight the diagnostic potential of T-thiol, Cu, and Zn levels in distinguishing AMI and HF. The determined cutoff values and associated sensitivity and specificity values provide valuable insights into the discriminatory power of these parameters. Further details are presented in Table 2.

**Table 2:** Zn and T-thiol ROC analysis results for AMI and HF groups, Cu ROC analysis results for HF and control groups.

Variable (s)	Risk Factor	AUC (95 %)	Cutoff value	p-value	Sensitivity ( %)	Specificity ( %)
T-thiol	AMI	0.669 (0.547‒0.790)	245.48	0.01	68.4	59
Cu	HF	0.796 (0.705‒0.886)	121.1	0.001	75.6	73.1
Zn	AMI	0.700 (0.594‒0.806)	84.50	0.01	68.8	63.8

Cu, Copper; T-thiol, Total Thiol; AUC, Area Under Curve; AMI, Acute Myocardial Infarction; HF, Heart Failure.

## Discussion

In the present study, TAS and Cu levels were increased in the disease groups compared to the control group. T-thiol, N-thiol, and Zn levels were increased in AMI compared to HF. The increase in disulfide level in the HF group compared to the AMI group suggests that disulfide level may be one of the parameters that should be evaluated after AMI. T-thiol and Zn levels may have the potential to be biomarkers for AMI, and Cu levels for HF.

TAS, one of the biomarkers used in the evaluation of oxidative stress, generally tends to decrease in cases where oxidative balance is impaired.^21^ However, unexpected increases in TAS levels have also been reported in the literature, especially in association with CAD.^22^ Indeed, Mahdy et al. found that TAS levels bi in AMI patient groups and suggested that this increase may be a result of compensatory mechanisms activated against increased oxidative stress.^23^ Consistent with this finding, TAS levels tended to increase in the patient groups compared to the control group in this study. Moreover, TAS levels were higher in the HF group compared to the AMI group, suggesting that a stronger or more prolonged antioxidant response develops to cope with oxidative stress in the chronic process. This suggests that despite the acute and rapid nature of AMI, the ongoing oxidative load in HF forces the organism to develop a more pronounced antioxidant defense. In addition, it has been reported that TOS and OSI values increased in AMI patients compared with the control group.^24^ In the present study, a similar trend of an increase in TOS and OSI levels was observed in the AMI group. This once again supports the role of increased oxidative stress in the pathophysiology of acute myocardial infarction.

The role of Zn in biochemical processes associated with oxidative stress has been the subject of curiosity in different studies. Saul et al. reported a relationship between Zn deficiency and susceptibility to oxidative damage.^25^ Liu et al. reported that serum Zn levels were significantly lower in AMI patients, and this decrease may be associated with acute tissue damage and increased risk of atherosclerosis.^26^ Moreover, the observed decline in Zn levels with increasing age-related risk of atherosclerosis^27^ suggests a potentially important relationship between Zn and atherosclerosis that requires further investigation. It has been reported that the decrease in serum Zn levels after AMI, which returns to normal in approximately 24 h, may be related to fluctuations in inflammation markers.^28^ In a study investigating the relationship between HF and Zn, it was found that serum Zn levels were lower in the HF group compared to the control group.^29^ These data suggest that changes in serum Zn levels in cardiovascular diseases such as AMI and HF should be considered in the context of oxidative stress. In this study, serum Zn levels were found to be lower in the HF group with an increasing trend in TOS values compared to the control group, in line with the expectations. However, remarkably, Zn levels were found to be higher in the AMI group with the highest TOS levels compared to the other groups. This suggests that more time-sensitive prospective studies are needed to determine whether the changes in Zn levels in AMI are a transient response specific to the acute phase of the disease or a more permanent imbalance.

Since changes in Zn levels can affect Cu concentrations and the cellular balance of Cu,^30^ A deeper understanding of the complex relationship between Cu and oxidative stress is extremely important. In the literature, it has been reported that a decrease in Zn leads to a decrease in the Zn/Cu ratio, increasing oxidative stress and thus increasing the prevalence of atherosclerosis.^31^ Epidemiological studies emphasise that Cu excess is associated with increased mortality in cardiovascular diseases, while Cu deficiency has similarly adverse effects.^32^ Considering that Cu, a heavy metal, participates in the structure of Cu-Zn SOD, Cu deficiency causes an increase in SOD, while Cu excess can cause peroxidative damage, especially in membrane lipids, and is therefore associated with CVD.^14^ There are also studies that found lower serum Cu levels in patients with Coronary Artery Disease (CAD) compared to controls.^33^ Studies have identified Cu as an independent risk factor for AMI, associated with increased oxidative stress and potential damage to the arterial endothelium.^34^ In the present study, serum Cu levels were significantly higher in both AMI and HF groups compared with the control group, which was consistent with previous findings.^35^ This increase in Cu levels, especially in AMI patients, may contribute to increased oxidative stress, as indicated by the observed high anti-oxidized LDL (anti-oxLDL) concentrations. The correlation between ceruloplasmin, a Cu-binding protein, and anti-oxLDL further supports the idea that Cu has a potential pro-oxidative role in cardiovascular diseases.^36^ These findings highlight the complex relationship between Zn-Cu homeostasis, oxidative stress, and the pathogenesis of cardiovascular diseases and provide valuable information about the underlying mechanisms that deserve further investigation.

The antioxidant effects of Zn and Cu are largely related to the binding of these elements to thiol groups that protect them against oxidation.^37^ Thiols containing a Sulfhydryl (SH) chain group act as antioxidants by stabilizing free radicals. When exposed to reactive oxygen species, SH groups undergo oxidation, leading to the formation of reversible disulfide bonds.^3^ Previous studies in individuals with CAD have shown an association between increased mortality and high cystine levels, emphasizing the importance of thiol balance.^38^ The present study demonstrates significantly higher T-thiol levels in the AMI group compared with both the control and HF groups, emphasizing the importance of thiol balance in CAD. In the context of these findings, it is noteworthy that Dunning et al.^39^ reported that high serum free thiol levels were associated with a reduced risk of cardiovascular mortality in the elderly population. Furthermore, the present observations align with those of Kundi et al.,^40^ who suggested that analyzing the disulfide/total thiol balance through ROC analysis could offer valuable insights in sera from AMI patients. Based on these views, the authors questioned the potential of serum T-thiol levels as a biomarker that can discriminate between AMI and HF. According to the ROC analysis results, serum Cu, Zn and T-thiol levels may be new biomarkers for the occurrence of AMI and the progression of AMI to HF.

Given the differences in the present study, multivariate linear regression analyses were conducted to determine whether the observed variations in serum Zn, Cu, TAS, and thiol levels remained significant after adjusting for age. The analyses revealed that zinc levels were significantly lower in the HF group compared to both the control and AMI groups, even after adjustment, suggesting a disease-specific alteration independent of aging. Although the group effects on TAS and thiol levels were attenuated following age adjustment, their directional patterns, particularly between the AMI and HF groups, remained consistent, implying distinct redox imbalances that may reflect underlying pathophysiological differences unrelated to age.

The present study findings focus on significant changes in serum Zn, Cu, and T-thiol levels related to oxidative stress in patients with AMI and HF. This suggests a potential effect of these trace elements on CAD pathophysiology. The results may guide future research by encouraging a more detailed examination of the effects of Zn and Cu on oxidative stress pathways and help to better understand their potential roles in CAD treatment.

## Limitations

This study has several limitations that should be acknowledged. Although these findings highlight significant associations between trace elements, thiol levels, and cardiovascular conditions, further research is necessary to confirm these results in broader populations. The lack of an established reference range for serum zinc, copper, and thiol levels in the context of cardiovascular disease continues to pose a challenge for clinical interpretation. Additionally, the age discrepancy between study groups may have influenced some outcomes despite statistical adjustment. Future studies with age-matched cohorts and larger sample sizes would enhance the generalizability and robustness of these findings.

## Conclusion

AMI and HF are common cardiovascular diseases characterized by elevated ROS production and a shift in the oxidative balance toward oxidants. Disturbances in Zn, Cu, and thiol levels not only affect each other but also affect the oxidative stress balance. This study represents a comparison of AMI and HF patients with each other and a control group in terms of the parameters examined. The findings of this study shed light on the complex relationships between Zn, Cu, thiol balances, and oxidative stress in CAD. Despite the modest AUC values in the ROC analyses, serum Cu, Zn, and T-thiol levels emerge as potential candidates as biomarkers in cardiovascular diseases. The unexpected findings in the present study highlight the complexity of these interactions, emphasizing the need for further research to unravel the nuanced mechanisms underlying oxidative stress in AMI and HF. In summary, while this study provides valuable insights, continued research efforts with expanded cohorts and refined methodologies will be important to elucidate the complex interactions and clinical implications of these factors.

## Availability of data and materials

The data that support the findings of this study are available from the corresponding author upon reasonable request.

## Consent for publication

The authors affirm that human research participants provided informed consent for publication of the images in Figs. 1a and b, 2a and b, 3a–c, 4–c, and Supplemental Fig. 1.

## Ethics approval and consent to participate

All ethical procedures have been approved by the Bezmialem Vakif University Non-Interventional Research Ethics Committee (2021/134, 03.05.2021). Informed consent was obtained from all individual participants included in the study.

## Authors’ contributions

Conceptualization and methodology: Y.A.C, B.T. and S.S. Data improvement and project management: Y.A.C, B.T. and S.S. Review and data analysis: Y.A.C., U.S. and N.A. Article writing-original draft: Y.A.C., U.S. and M.D. This manuscript has been read and approved by all authors, and each author believes that the manuscript represents honest work.

## Funding

This work was supported by the Bezmialem Foundation University Scientific Research Projects Unit (Project number: 20210609).

## Declaration of competing interest

The authors declare no conflicts of interest.

## Appendix Supplementary materials

Image, application 1
