# Supplementary material for: Assessment of oxidative stress and trace element dynamics in acute myocardial infarction and heart failure: a focus on zinc, copper, and thiol dynamics
Source: Clinics (Sao Paulo). 2025 Aug 21;80:100755. doi: 10.1016/j.clinsp.2025.100755 (PMC12398269; doi:10.1016/j.clinsp.2025.100755)

CLINICS-D-25-00616_Supplementary Material

**Supplemental Figure 1** Clustering results are visualized as a heatmap, with the distance measure using Euclidean and the clustering algorithm using Ward. D. Abbreviations: T-thiol, Total Thiol; N-thiol, Native Thiol; TAS, Total Antioxidant Status; TOS, Total Oxidant Status; OSI, Oxidative Stress Index; C, Control; AMI, Acute Myocardial Infarction; HF, Heart Failure.


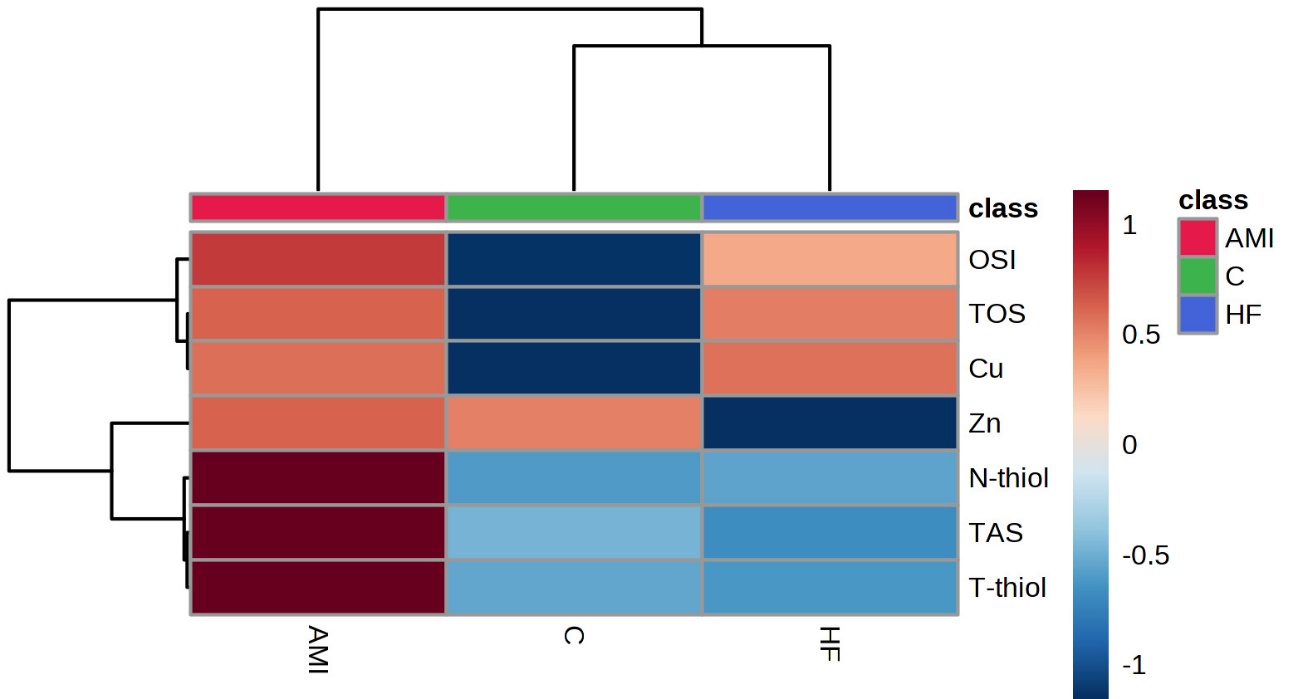

Supplement: Supplementary file 1 [file mmc1.docx]
